# Scoping Review of Clinical Presentations and Outcomes in Patients with Concomitant COVID-19 Infection and Acute Mesenteric Ischaemia

**DOI:** 10.3390/v16040506

**Published:** 2024-03-26

**Authors:** Wenyi Cai, Yi Zhao, Sreelakshmi Mallappa

**Affiliations:** 1 East Suffolk and North Essex Foundation Trust, Colchester CO4 5JL, UK; 2 Colchester General Hospital, Turner Road, Colchester CO4 5JL, UK; 3 Imperial College London School of Medicine, London SW7 2DD, UK; yi.zhao18@imperial.ac.uk; 4 West Hertfordshire Teaching Hospitals NHS Trust, Hertfordshire WD18 0HB, UK; sreelakshmi.mallappa2@nhs.net; 5 The Hillingdon Hospitals NHS Foundation Trust, Uxbridge UB8 3NN, UK

**Keywords:** COVID-19, thromboembolism, mesenteric ischaemia

## Abstract

Objectives: COVID-19 infection confers an increased risk of coagulation dysfunction (1) predisposing to thromboembolism in many anatomical sites including the gastrointestinal tract (GIT) (2). This study investigates the clinical presentation and outcome in patients presenting with concurrent COVID-19 infection and gastrointestinal tract ischaemia. Furthermore, differentiation and comparisons are drawn between those with arterial and venous aetiology for mesenteric ischaemia. Methods: A systematic search was undertaken on EMBASE, PubMed, and MEDLINE. Two independent reviewers screened titles, abstracts, and full-text articles according to the inclusion criteria and extracted relevant data. Data analyses were conducted using Excel^®^. Results: Forty-one studies were included in the data analyses, yielding 44 patients. Twenty-six patients had mesenteric arterial occlusion, sixteen patients had mesenteric venous occlusion, and two patients had both arterial and venous mesenteric occlusion. All patients had concurrent COVID-19 infection. The survival rate in patients with arterial aetiology was 38.5% in contrast to 68.8% in patients with venous aetiology. Twelve patients (29.3%) experienced respiratory symptoms in the community before the onset of gastrointestinal symptoms, and five (12.2%) developed gastrointestinal symptoms during their inpatient stay for COVID-19 pneumonitis. Conclusions: Acute mesenteric ischaemia presents a clinical challenge to diagnose due to its non-specific symptoms. Concurrent COVID-19 infection with its predominant respiratory symptoms adds a further challenge in recognising the non-specific symptoms of mesenteric ischaemia. Our study draws attention to the increased thromboembolic risk posed by COVID-19 infection and the need for a high index of suspicion to aid prompt diagnosis and management of acute mesenteric ischaemia, even in the post-pandemic era.

## 1. Introduction

The novel severe acute respiratory syndrome coronavirus 2 (SARS-CoV-2) emerged in Wuhan, China in December. It quickly spread worldwide and was declared a pandemic by the World Health Organisation (WHO) in March. SARS-CoV-2 causes coronavirus disease 2019 (COVID-19), which predominantly affects the respiratory tract, but it can have a broad spectrum of presentations ranging from being asymptomatic to multi-organ dysfunction [1].

In patients with complicated COVID-19 infections needing acute hospital admission, it has been well-established that these patients are at an increased risk of thromboembolism [2,3,4]. Knight et al. refined the study of thromboembolism risk after COVID-19 infection into arterial and venous thrombosis [5]. It was found that the adjusted hazard ratios were higher in both groups when compared to their respective non-COVID-19 cohort [5]. Coagulation dysfunction in patients with COVID-19 is reflected through elevated markers of coagulability, such as high levels of D-dimer and prolonged prothrombin time [6]. The binding between SARS-CoV-2 and its host receptor, angiotensin-converting enzyme 2 (ACE2), upregulates the expression of tissue factors on cell surfaces. This activates the extrinsic coagulation pathway, sending the human body into a prothrombotic state [7,8]. In addition, it has been shown that there is an influx of proinflammatory cytokines and chemoattractants in circulation during COVID-19 infection, especially in the case of a severe infection [9,10]. This induced cytokine storm can cause extensive endothelial injury, thus activating the process of haemostasis [11]. An existing observational study has outlined the relationship between severe COVID-19 infection and the increased thrombosis risk [12].

Multiple studies have shown that the most common manifestations of thromboembolism in hospitalised COVID-19 patients are deep vein thrombosis and pulmonary embolism, and the risk increases further if the patient is admitted to an intensive care unit [3,4,13]. Although thromboembolism in mesenteric vessels is rare, it has been reported in COVID-19 patients in several case series [14,15,16]. Acute mesenteric ischaemia (AMI) is defined as a sudden disruption in circulation to the intestines necessitating urgent intervention in most cases to preserve the viability of the bowel [17]. The aetiology of AMI can be attributed to the occlusion of mesenteric arteries and mesenteric veins due to an embolus or a thrombus. In many cases, vasoconstriction of mesenteric vessels can interrupt blood supply without intraluminal occlusion, leading to non-occlusive AMI [17,18,19].

The current literature on COVID-19-induced AMI has looked into patient presentations and established a high mortality rate in these patients [20,21,22]. A further subset of studies investigated the radiological signs of AMI during COVID-19 and highlighted the importance of cross-sectional imaging to diagnose AMI promptly [16,23,24,25]. However, there is limited research on the effect different types of AMI have on patient outcomes during COVID. This study aims to conduct a scoping review of outcomes in patients with concurrent COVID-19 and AMI, considering the classifications of AMI.

## 2. Methods

The Preferred Reporting Items for Systematic Reviews and Meta-Analyses extension for scoping reviews (PRISMA-ScR) were followed [26]. The protocol for this study has been previously published [27].

### 2.1. Research Question

What is the impact of aetiological differences of AMI on the outcomes in COVID-19 patients?

### 2.2. Search Strategy

A systematic search was undertaken in EMBASE, PubMed, and Medline. The full search strategy was: (“Coronavirus disease” OR “coronavirus infections” OR “COVID-19” OR “2019-nCoV” OR “SARS-CoV-2”) AND clinical AND (“Intestinal isch*” OR “bowel isch*” OR “mesenteric isch*” OR “thromb*”) AND Imaging. This was adapted for each included database. There were exchangeable terms describing the location of acute mesenteric ischaemia, such as “bowel” and “intestinal”. Moreover, multiple terms describing the loss of blood supply to the bowel, such as “embolism”, “thrombosis”, “thromboembolism”, and “ischaemia” were collectively analysed. All included articles’ reference lists were screened for additional studies. Studies published in the English language from November 2019 to date were included.

### 2.3. Eligibility Criteria

Studies describing adult COVID-19 patients diagnosed with clinical manifestation and radiologically confirmed bowel ischaemia were included. Included studies also indicated the site of pathology from the radiological reports. Only studies published in the English language were included. Paediatric populations and patients with thromboembolic events in other organs, such as myocardial infarction, stroke, and pulmonary embolism, were excluded. Studies discussing other coronaviruses were excluded.

The study’s aim was to investigate the distribution of patient demographics and clinical symptoms of AMI with arterial, venous, and non-occlusive aetiology.

### 2.4. Study Selection Procedure

Extracted studies were uploaded to Rayyan for the organisation of the screening process [28]. Two independent reviewers (W.C. and Y.Z.) screened through the returned searches by evaluating the title and abstract according to the inclusion criteria. The selected studies were compared between the reviewers, any discrepancies were discussed, and a third reviewer (S.M.) was sought if agreement could not be reached. Full-text articles were accessed for studies that met the inclusion criteria. Upon studying full-text articles, only studies that specified the aetiology of AMI by obtaining cross-sectional imaging were included in the final study selection; our study aim necessitated this.

### 2.5. Data Extraction

Information on patient demographics was extracted from each study, including sex and age. The following clinical data were extracted: patient comorbidities, presenting complaints, imaging findings, and management plans. All data were collated and analysed in Microsoft Excel version 16.83^®^ [29].

## 3. Results

**Figure 1:**
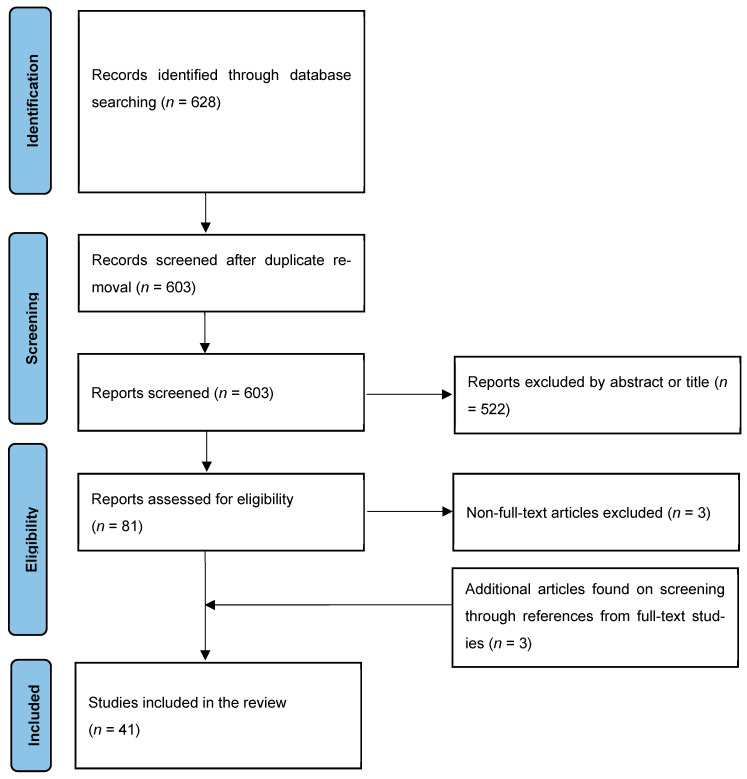
PRISMA flow diagram of evidence acquisition. PRISMA = Preferred Reporting Items for Systematic Reviews and Meta-Analysis.

The database search yielded 628 articles. After removal of duplicates and screening of the title and abstract, 81 studies remained. These studies underwent full-text evaluation, and a further three studies were retrieved upon screening these studies’ references. Forty-one studies reporting on 44 patients were included in the final selection [14,16,30,31,32,33,34,35,36,37,38,39,40,41,42,43,44,45,46,47,48,49,50,51,52,53,54,55,56,57,58,59,60,61,62,63,64,65,66,67,68]. Of these, 32 were case reports, and the remaining 9 were case series. Figure 1 demonstrates the PRISMA flow diagram of this study. Table 1 shows the summaries of the included studies.

The mean age of patients in the study selection was 55.6 (±16.9) years, and 26 out of 44 (59%) patients were male. Table 2 shows a summary of the studies and the patient demographics.

The most common presenting complaints on admission were abdominal pain (77.3%) and vomiting (38.6%). Five patients presented with solely respiratory symptoms, and all developed abdominal pain later during their admission [43,53,54,65,66]. Thirteen patients who tested positive for COVID-19 made contact with healthcare services before presenting to the emergency department with gastrointestinal symptoms [16,30,31,32,35,37,38,40,41,45,48,54,63]. All patients had cross-sectional imaging to confirm the diagnosis of acute mesenteric ischaemia.

### 3.1. Arterial Mesenteric Ischaemia

Twenty-six patients (59.1%) had exclusively arterial mesenteric ischaemia. Twenty-three of them were found to have isolated superior mesenteric artery (SMA) occlusion, one patient had occlusion in both the coeliac trunk and the SMA, and the remaining two patients were found to have occlusion in the branches of the SMA. Twenty patients in this cohort underwent laparotomy, two underwent endovascular treatment, and one patient was deemed not a candidate for surgical treatment. The mean length of stay was 29.4 days amongst five reported cases. The survival rate for this group was 38.6%.

### 3.2. Venous Mesenteric Ischaemia

Sixteen patients (36.4%) had exclusively venous mesenteric ischaemia, eleven of them had isolated occlusion in the superior mesenteric vein (SMV), and the remainder had involvement of other mesenteric veins and the portal vein. Eight patients underwent laparotomy, and one patient was deemed unsuitable for surgical intervention. The survival rate for this group was 68.8%.

### 3.3. Mixed Arterial and Venous Mesenteric Ischaemia

Two patients were found to have mixed arterial and venous mesenteric ischaemia. Both underwent exploratory laparotomy, and one patient survived.

### 3.4. Anticoagulation Management

Eleven patients among twenty-six patients with arterial mesenteric ischemia received either anticoagulation treatment or a combination of anticoagulants and antiplatelets. Twelve patients among sixteen patients with venous mesenteric ischaemia received anticoagulation treatment. One patient out of two with mixed arterial and venous thrombosis received anticoagulation treatment. Fourteen patients out of twenty-four patients (58.3%) who received anticoagulation treatment survived. Five patients’ survival statuses after receiving anticoagulation were not reported.

## 4. Discussion

This study has investigated the presentations and outcomes in patients with COVID-19 and AMI. We found that the majority of patients had arterial mesenteric ischaemia provoked by COVID-19 infection, and the survival rate was better among patients in the venous mesenteric ischaemia group compared to those with arterial mesenteric ischaemia.

Gastrointestinal symptoms are widely prevalent in patients with COVID-19 [69,70]. It has been observed that diarrhoea is a common symptom during the early course of infection [71]. These findings contrast this study, where abdominal pain was the most typical gastrointestinal symptom. In addition, it has been postulated that gastrointestinal symptoms may indicate or predict a more severe case of COVID-19 [69,72].

This study highlights that respiratory symptoms and abdominal symptoms do not always present at the same time. Many patients had sought medical attention for respiratory symptoms days or weeks before the onset and subsequent severity of abdominal pain necessitated their emergency department visit. It may be common for clinicians to treat the two clusters of symptoms as separate disease entities, leading to delays in diagnosis.

Aetiology of AMI can be either due to an embolus or thrombosis. The clot leading to the occlusion of mesenteric vessels can be an embolus or worsening of existing thrombosis [17]. It has been estimated that half of AMI cases are due to SMA embolus [73,74]. This finding corresponds with this study, where more than half of the patients had an isolated SMA occlusion, but it is unknown if these occlusions were of embolic or thrombotic origin. Whilst patients with AMI secondary to an embolus in SMA typically present with worsening acute abdominal pain, in contrast, patients with AMI secondary to thrombosis of SMA typically have a background history suggestive of chronic mesenteric ischaemia; associated symptoms are postprandial abdominal pain and fear of eating [17].

Another way to classify AMI is to differentiate between arterial and venous pathology. Mesenteric venous ischaemia is less common than mesenteric arterial ischaemia [18]. Mesenteric venous ischaemia tends to affect a younger population, and any congenital hypercoagulable condition should be investigated and ruled out as a potential cause [18].

AMI can also be caused by nonocclusive aetiology. Non-occlusive mesenteric ischaemia is due to vasoconstriction of the mesenteric vessels without an intraluminal clot [75]. It accounts for approximately 20% of cases, and it has been observed in critically unwell patients in an intensive care setting [75].

Computed tomography angiography (CTA) of mesenteric vessels is essential in the timely diagnosis of AMI and in the differentiation between different types of AMI, primarily due to the lack of laboratory tests available to accurately diagnose mesenteric ischaemia as a differential diagnosis [17].

Anticoagulation is strongly recommended for all cases of AMI from the time of diagnosis. However, the indication is more robust in the case of venous mesenteric ischaemia than in the other types [17,18,76,77]. Extended anticoagulation through post-operative and post-discharge is crucial to reduce the incidence of future clots.

Once AMI is confirmed, open surgery should be performed promptly to assess bowel vitality [17]. The endovascular approach has become increasingly favourable when the facility allows for achieving revascularisation [78].

Mesenteric ischaemia has a mortality rate of more than 50% [79,80]. Delay in diagnosis is a significant factor contributing to the high mortality rate—only five studies reported the time lapse between patient presentation and diagnosis in this study.

### Limitations

As COVID-19 is still a relatively new disease and AMI is a rare differential diagnosis of abdominal pain, studies with methodologies that occupy a higher level in the hierarchy of evidence are currently lacking in number. Thus, inferences cannot be drawn to the general population regarding prevalence, risks, and hazard ratios, and causal relationships cannot be established by looking at case studies. Furthermore, the information reported in each case report or case series differs widely, resulting in possible gaps in our data. Finally, other more common contributing factors of AMI other than COVID-19 infection, such as atrial fibrillation and other cardiac causes of embolism, have not been adequately investigated in the selected studies.

## 5. Conclusions

Acute mesenteric ischaemia is challenging to diagnose due to its low prevalence and non-specific symptoms. COVID-19’s predominant respiratory symptoms may drive the diagnostic focus away from considering AMI as a differential diagnosis. This study has shown poor survival outcomes in patients with concurrent COVID-19 infection and AMI. With emerging, new strains of COVID-19, the disease is ever-evolving, prompting a much higher index of suspicion, which aids in the prompt diagnosis and management of this life-threatening surgical emergency in the post-pandemic era.

## Figures and Tables

**Table 1:** Summary of final selection of studies including first author, year of publication, number of patients, type of study, and aetiology of AMI.

References	Year of Publication	Number of Patients	Type of Study	Aetiology of AMI
Abeysekera et al. [30]	2020	1	Case report	Venous
Ahmed et al. [31]	2020	1	Case report	Venous
Alali et al. [32]	2021	1	Case report	Arterial
Alemán et al. [33]	2021	1	Case report	Venous
Amaravathi et al. [34]	2021	1	Case report	Arterial and venous
Aryal et al. [35]	2023	1	Case report	Arterial
Azouz et al. [36]	2020	1	Case report	Arterial
Bannazadeh et al. [37]	2021	1	Case report	Arterial
Beccara et al. [38]	2020	1	Case report	Arterial
Calcagno et al. [39]	2021	1	Case report	Venous
Cheung et al. [40]	2020	1	Case report	Arterial
Costa et al. [41]	2022	1	Case report	Arterial
De Barry et al. [42]	2020	1	Case report	Arterial and venous
Dinoto et al. [43]	2021	1	Case report	Arterial
Etkin et al. [14]	2021	2	Case series	Arterial
Fan et al. [44]	2020	1	Case report	Venous
Filho et al. [45]	2020	1	Case report	Venous
Fransvea et al. [46]	2022	2	Case series	Arterial
Goodfellow et al. [47]	2021	1	Case report	Venous
Gupta et al. [48]	2023	1	Case report	Arterial
Hoyo et al. [49]	2020	1	Case report	Venous
Hussein et al. [50]	2021	1	Case report	Venous
Ignat et al. [51]	2020	1	Case series	Venous
Jeilani et al. [52]	2021	1	Case report	Venous
Karna et al. [53]	2020	1	Case report	Arterial
Khaleghi et al. [16]	2021	1	Case series	Arterial
Krothapalli et al. [54]	2021	1	Case report	Arterial
Lari et al. [55]	2020	1	Case series	Venous
Mahruqi et al. [56]	2021	1	Case series	Arterial
Mitchell et al. [57]	2021	1	Case report	Arterial
Nada et al. [58]	2022	1	Case report	Arterial
Nasseh et al. [59]	2021	1	Case report	Arterial
Norsa et al. [60]	2020	1	Case report	Venous
Osilli et al. [61]	2020	1	Case series	Arterial
Pang et al. [62]	2021	1	Case report	Venous
Posada-Arango et al. [63]	2021	2	Case series	Arterial
Rodriguez-Nakamura et al. [64]	2020	1	Case series	Venous
Sukegawa et al. [65]	2022	1	Case report	Arterial
Ucpinar et al. [66]	2020	1	Case report	Arterial
Vidali et al. [67]	2021	1	Case report	Venous
Vulliamy et al. [68]	2020	1	Case report	Arterial

**Table 2:** Patient characteristics of the included cases stratified by arterial, venous, and mixed aetiology.

	Arterial	Venous	Mixed (Arterial + Venous)
***Demographics***			
Number of patients	26	16	2
% Female	26	36	50
Mean age	64	43	62
***Comorbidities***			
Chronic kidney disease	2	0	0
Heart failure	1	0	0
Atrial fibrillation	3	0	0
Ischaemic heart disease	2	0	0
Hypertension	9	2	0
Diabetes mellitus	4	2	0
***Presenting complaints***			
Abdominal pain	18	16	2
Fever	6	1	1
Diarrhoea	5	2	1
Abdominal distension	2	1	0
Vomiting	10	7	1
Cough	5	0	0
Breathlessness	5	2	1
***Laboratory parameters***			
White cell count (10^9^/L)	15.5	18.0	12.6
C-reactive protein (mg/L)	180.8	184.4	125
Lactic acid (mmol/L)	4.4	1.6	5.4
***Management***			
No treatment	3	2	0
Anticoagulation only	1	2	0
Surgery only	6	3	1
Anticoagulation + surgery	9	4	1
***Outcomes***			
Survived	10	7	1
Deceased	9	4	1
Unknown	7	5	0

## Data Availability

Raw data can be accessed upon personal request.
